# A computational study of energy barriers of structural transformations and hydrogen transfer in boehmite

**DOI:** 10.1039/c7ra12273j

**Published:** 2018-01-09

**Authors:** Yingjian Jiang, Yaoping Xie, Haibo Guo

**Affiliations:** School of Materials Science and Engineering, Shanghai University Shanghai China guohaibo@shu.edu.cn

## Abstract

The crystal structure of boehmite (γ-AlOOH) contains a large amount of hydrogen bonds that are joined into chains by sharing hydrogen-bond donor and acceptor oxygen atoms. The hydrogen ions in the hydrogen-bond chains are highly mobile and have complicated structural characterizations, and this feature may well be utilized for proton-conducting applications, but the mechanism is unknown without the dynamic parameters of the hydrogen-transfer processes. We propose probable hydrogen-transfer paths and compute their energy barriers using density functional theory with van der Waals density functionals, on both perfect and vacancy-containing crystal structures. It is revealed that the energy barriers are generally below 21 kJ mol^−1^ in a perfect crystal, and 14 kJ mol^−1^ in a vacancy-containing structure. The low energy barriers are indicators of the high proton conductivity of boehmite even at room temperature.

## Abstract 2

Energy barriers for hydrogen-transfer processes are generally below 21 kJ mol^−1^ in a perfect crystal, and 14 kJ mol^−1^ in a vacancy-containing structure. The low energy barriers are indicators of the high proton conductivity of boehmite.

## Graphical abstract

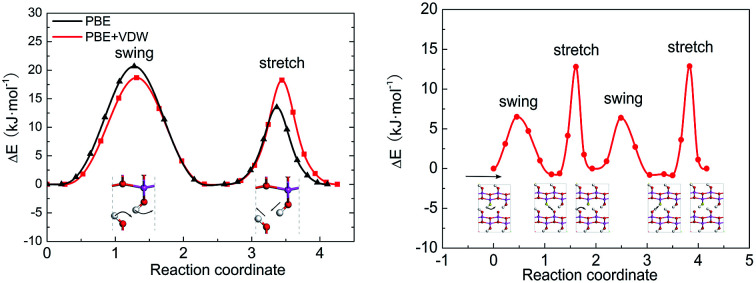

## 1. Introduction

Boehmite (γ-AlOOH) is an aluminum oxyhydroxide, metastable relative to diaspore (α-AlOOH) under ambient conditions. Boehmite occurs naturally as one of the main components of bauxite ore,^1,2^ and has been studied by various research groups for multiple applications, including catalysts,^3^ adsorbents,^4–7^ and thin porous membranes.^8^ For these applications, boehmite is frequently used as a precursor for manufacturing alumina phases, such as γ-Al_2_O_3_ and α-Al_2_O_3_, usually through thermal dehydration.^9^ Less explored, however, is that boehmite itself may serve as a proton conductor for electrochemical reactions in aqueous environments.

Recently, a particularly large amount of work has been focused on proton-conducting materials,^10^ which have many applications in the electrochemical industry. For instance, proton exchange membrane (PEM) fuel cells (PEMFC) rely critically on proton conductors, in form of thin membranes, for charge and mass transport. The general requirements of a good electrolyte membrane for PEMFCs are: high proton conductivity (>10^−2^ S cm^−1^), good chemical stability in highly acidic media and thermal stability at low to medium temperatures, low cost, and ability to be processed into thin films.^11^ Many PEMs have been developed, some being commercialized, but no membranes meet all the criteria. Commercially available products like Nafion developed by Dupont de Nemours also have some limitations, among which the most serious one is that proton conductivity drops significantly if operating temperature is above 90 °C.^12,13^ Therefore, finding alternative materials to improve or replace currently available PEMs is an important aim of numerous researches about PEMs.

Previous studies have revealed that hydrogen bonding plays an important role in proton conduction mechanisms.^14,15^ Thus, those structures that contain a large amount of hydrogen bonds are likely good proton conductors. Boehmite has a similar hydrogen-bond network as water, which was deemed to be an excellent proton conductor.^16^ It is of technological interest to investigate the proton-conducting ability of the hydrogen-bond network in boehmite. Since boehmite dehydrate at about 400 °C (variable with samples and moisture),^17^ much higher than that of boiling temperature of bulk water under ambient pressure, it is possible to prepare proton exchange membranes that can work at higher temperatures than current Nafion membranes. Elevated working temperatures in the context of PEMFCs mean improved reaction kinetics of the electrochemical reactions, and reduced chance of catalyst poisoning. Other advantages of boehmite include its low cost and well-developed synthesis methods. With all these advantages, the proton conductivity of boehmite, however, has seldom been measured, partly because boehmite is highly anisotropic and protons may only diffuse along a specific direction.

In boehmite's crystal structure, hydrogen-bonds connect to one another and form chains along crystallographic [001] direction of the orthorhombic cell. These parallel hydrogen-bond chains bind interlocking double layers of edge-sharing AlO_6_ octahedra that are stacked along [010] direction. The locations of Al and O atoms in boehmite have been well measured by X-ray diffraction (XRD) and neutron diffraction experiments.^18,19^ The locations of H atoms in boehmite, however, have been elusive for a long time in spite of multiple structural characterizations based on Raman,^20,21^ infrared (IR)^22^ and nuclear magnetic resonance (NMR) spectroscopy.^23^ Because H atoms are weak X-ray scatters, it is difficult to locate the H atoms in boehmite from XRD experiments. The crystal structure of boehmite was deduced to be in the space group *Cmcm* from the definite positions of Al and O atoms. The neutron diffraction technique, which has much higher resolution in measuring H atoms' positions than XRD, confirms the structural characterization results by XRD. The previous researches on synthetic lepidocrocite (γ-FeOOH)^24,25^ also attributed the structure to the *Cmcm* group.

In the unit cell of boehmite, two probable positions of hydrogen atoms are found to be compatible with the space group *Cmcm* in boehmite. The hydrogen atoms may reside in Wyckoff site 8*e*, forming O–H bonds that are perpendicular to the double-sheet planes. This is highly unlikely because it doesn't conform to the oxygen's orthogonal bonding structure, and researchers have not found significant electron density at this position.^26^ Ewing^25^ proposed that the hydrogen atom is located in Wyckoff site 4*a*, the midpoint tween two interlayer oxygen atoms. By this way, symmetric O–H–O hydrogen bonds are formed. This structure is, however, not acceptable because of the exceptionally long bond length of the symmetric O–H–O bonds, approximately 2.67 Å, which is apparently larger than 2.55 Å for most of confirmed symmetric O–H–O bonds.^27^

Several space groups conforming to asymmetric O–H⋯O hydrogen bonds have been considered, such as *Cmc*2_1_,^28^*Pca*2_1_ and *P*2_1_/*c*.^29^ The only real difference among these structures is the relative positions of the H atoms. Most of previous studies focus on relative energetic stabilities of the probable crystal structures of boehmite. Some questions, like whether the above crystal structures can transform into each other and how defects affect the structural transformations, are unsolved. Hence, we address the structural transformations for both perfect crystal structure and crystal structures with defects. In the first part, we investigate the *Cmc*2_1_ and *Pca*2_1_ space groups, as proposed by previous studies, and propose a new space group of *Pmc*2_1_ for boehmite. For the second part, we focus on the defects of hydrogen vacancies for simplicity. The structural transformations involve breakage and formation of O–H bonds in the hydrogen-bond chains, so we calculate energy barriers for these processes to assess the easiness (or difficulty) of the transformations.

These short-distance displacements of hydrogen atoms in boehmite, when combined with the long hydrogen-bond chains, may result in long-distance displacement of the hydrogen atoms, following the Grotthuss hopping mechanism.^30^ By calculating the energy barriers for the structural transformations, we are able to estimate proton conductivity as the two properties are tightly correlated. Proton exchange membranes are primarily characterized by proton conductivity.^31^ Before extensive experimental measurement of the proton conductivity, it is very useful of computational studies to assess probable mechanisms of hydrogen transfer in this highly anisotropic material.

## 2. Methodology

In this study we investigate thermodynamic stability of boehmite in space groups *Cmc*2_1_ (space group no. 36),^28^*Pmc*2_1_ (space group no. 26), and *Pca*2_1_ (space group no. 29)^29^ by comparing their total energies. Energy barriers associated with proton-transfer paths are calculated using the climbing-image nudged elastic band (CI-NEB) method implemented in VTST code.^32,33^ All the calculations are done using density functional theory (DFT) implemented in Vienna *Ab initio* Simulation Package (VASP).^34–36^ The electronic exchange and correlation interactions are described by the PBE (Perdew–Burke–Ernzerhof) functionals,^37^ and van der Waals density functionals (vdW-DF)^38–40^ combined with exchange part of PBE, referred to as vdW-PBE hereafter. The effects of nuclei and core electrons are described using the Projector Augmented Wave (PAW) method.^41,42^ The PAW datasets for elements Al, O, and H are from the PAW dataset library shipped with VASP. The core radii are 1.90 Bohr for Al, 1.52 Bohr for O, and 1.10 Bohr for H.

As for computational settings, the cut-off energy of plane wave functions is 500 eV, electronic state occupations are modeled using the Gaussian smearing method with a small smearing width of 0.005 eV, and total energies are extrapolated to zero electronic temperature. Sampling in the first Brillouin zone is done on special *k*-points generated according to the Monkhorst–Pack method^43^ using a regular grid of 8 × 8 × 6 for the primitive cell of the *Cmc*2_1_ structure, 10 × 6 × 8 for the *Pmc*2_1_ structure, and 6 × 6 × 8 for the *Pca*2_1_ structure, according to tests by a convergence criterion of 0.001 eV per formula unit. The numbers of irreducible *k*-points are 60, 60, and 36 for the *Cmc*2_1_, *Pmc*2_1_, and *Pca*2_1_ structures, respectively. The convergence criteria are set to 1.0 × 10^−7^ eV for energy differences in self-consistent-field iterations, and 0.005 eV Å^−1^ for residual Feynman–Hellmann forces in relaxations.

Structures with hydrogen vacancies are generated by removing one hydrogen atom from 2 × 1 × 2 and 3 × 1 × 2 supercells with respect to the conventional cell of *Cmc*2_1_. We use two supercells (instead of one) to check the interactions between vacancies of image cells in the periodic boundary setting. The grids for generating *k*-points in the Monkhorst–Pack scheme are 8 × 4 × 8 for the 2 × 1 × 2 supercell, and 4 × 2 × 8 for the 3 × 1 × 2 supercell, resulting in 64 and 16 irreducible *k*-points, respectively.

For the transition-state calculations, we create 4 images between the starting and end structures by linear interpolations using a utility program of the VTST scripts. Our tests with 8 and 16 images for the transition from *Cmc*2_1_ to *Pmc*2_1_ show that the calculated energy barriers are already converged within 0.005 eV for the calculations with 4 images. The force-based quick-min algorithm is used for finding minimum-energy path. Cell parameters are kept constant for all the transition states.

## 3. Results and discussion

### 3.1 Crystal structures without defects

**Fig. 1:**
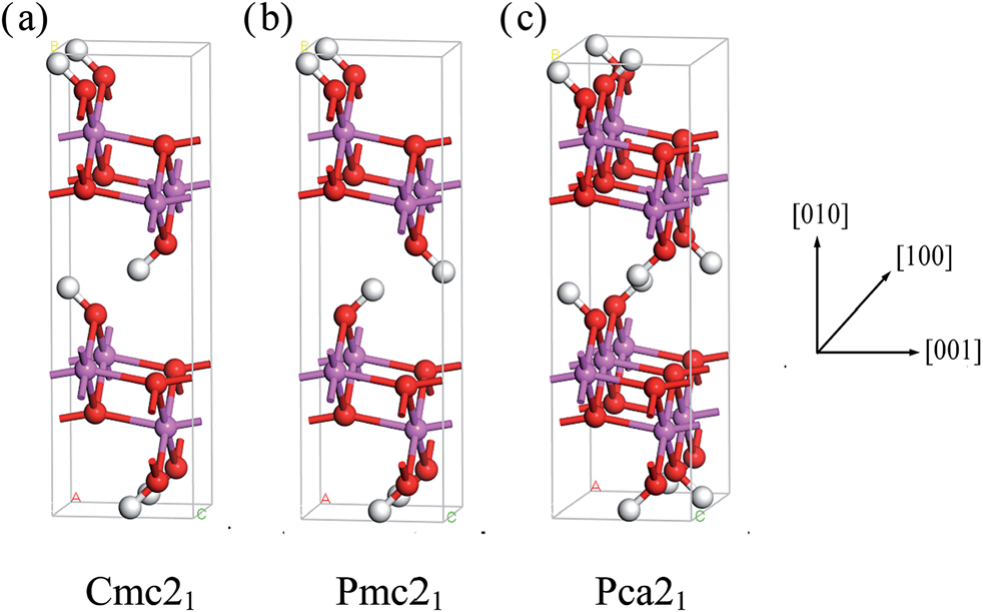
Crystal structures of boehmite oriented along the [100] direction. White atoms are H, red atoms are O, and pink atoms are Al. The structure in (a) is of *Cmc*2_1_ symmetry, (b) is of *Pmc*2_1_, and (c) is of *Pca*2_1_ space groups.

We can clearly see that boehmite has a layered structure (see Fig. 1) that contains AlO_6_ octahedron layers and hydrogen-bond layers. Each hydrogen-bond layer is composed of parallel hydrogen-bond chains along the [001] crystallographic direction. The boehmite crystal structures of *Cmc*2_1_, *Pmc*2_1_ and *Pca*2_1_ space groups are all orthorhombic, and the main difference is orientations of the hydroxyl groups. Since around each oxygen atom in the hydrogen-bonding layers there are two positions at which a hydrogen atom may reside, corresponding to two possible configurations for a hydrogen-bond chain. For the *Cmc*2_1_ structure, all the hydroxyl groups (O–H) tilt towards the [001̄] direction; for the *Pmc*2_1_ structure, the hydroxyl groups switch directions in adjacent hydrogen-bond layers; and for the *Pca*2_1_ structure, the hydroxyl groups in adjacent hydrogen-bond chains in a same layer have opposite directions.

The calculated lattice parameters of the *Cmc*2_1_ structure are listed in Table 1, together with experimental values. By comparing the lattice parameters from plain PBE and vdW-PBE calculations, we find that the values for *a* are almost the same (less than 0.01 Å or 0.3% in differences) in both settings, the values for *c* are also almost identical, and both are larger than the experimental values by about 1.1%. As for the lattice parameter *b* that is pertinent to interlayer distances, the value calculated by vdW-PBE is slightly smaller than the experimental value by about 0.01 Å, while the value by PBE differ from experimental value by about 0.1 Å. In general, vdW-PBE performs better in reproducing the lattice parameters of boehmite.

**Table 1:** Cell parameters of the *Cmc*2_1_ structure. Each orthogonal cell contains 4 formula units (*Z* = 4). Unit: Å

Reference	*a*	*b*	*c*
This work; PBE	2.8946	12.1465	3.7361
This work; vdW-PBE	2.9021	12.2224	3.7325
Experimental^44^	2.8681	12.2336	3.6923

It is noted that the orthorhombic cell of *Pca*2_1_ is twice that of *Cmc*2_1_ and *Pmc*2_1_. The total energies of the three structures, *i.e.*, *Cmc*2_1_, *Pmc*2_1_, and *Pca*2_1_, are compared to sort their relative thermodynamic stability. From Table 2 we can see that the *Pmc*2_1_ structure has almost identical total energy as *Cmc*2_1_ in both PBE and vdW-PBE calculations, but *Pca*2_1_ has consistently lower total energy than *Cmc*2_1_ and *Pmc*2_1_, by −0.27 kJ mol^−1^ in PBE or −0.26 kJ mol^−1^ in vdW-PBE calculations. Such small energy differences are close to resolution limits of experimental measurements and computations. The difference should be treated carefully because even though it is not negligible, the value is one order of magnitude smaller than the thermal fluctuation energy at room temperature (about 2.5 kJ mol^−1^). Thus, we show that the thermodynamic stability of *Cmc*2_1_ and *Pmc*2_1_ are essentially the same, and *Pca*2_1_ is slightly more stable than *Cmc*2_1_ and *Pmc*2_1_.

**Table 2:** Total energies of the *Pmc*2_1_ and *Pca*2_1_ structures with respect to the *Cmc*2_1_ structure. Unit: kJ mol^−1^

Method	*Pmc*2_1_	*Pca*2_1_
PBE	<0.01	−0.27
vdW-PBE	<0.01	−0.26

The order of thermodynamic stability of the three structures is related to the orientation of hydroxyl groups in hydrogen-bond layers. In *Cmc*2_1_ and *Pmc*2_1_, all hydroxyl groups in a hydrogen-bond layer point to a same direction (either [0  0 1] or [0 0 1̄]), and have the same polarity. In *Pca*2_1_, hydroxyl groups in a hydrogen-bond layer point to opposite directions in alternating chains, and collectively have zero polarity. Interactions between electric dipoles of a same polarity increases the system's energy, while interactions between electric dipoles of opposite polarities are associated with a negative energy. It appears that dipolar interactions are negligible across neighboring hydrogen-bond layers, since the energies of *Cmc*2_1_ and *Pmc*2_1_ are almost identical. The interactions of dipole moments in adjacent chains in a same layer should be very weak, at the order of 0.1 kJ mol^−1^ that is readily overridden by thermal fluctuations at room temperature. We conclude that the three structures have the same thermodynamic stability within an error of 0.5 kJ mol^−1^, and tentatively propose that hydroxyl polarity contributes slightly to their relative thermodynamic stability. In a same layer, the orientation of the hydroxyl groups in different zigzag-shaped chains should be independent of each other at normal conditions. This conclusion is consistent with a previous report in the literature.^26^

**Fig. 2:**
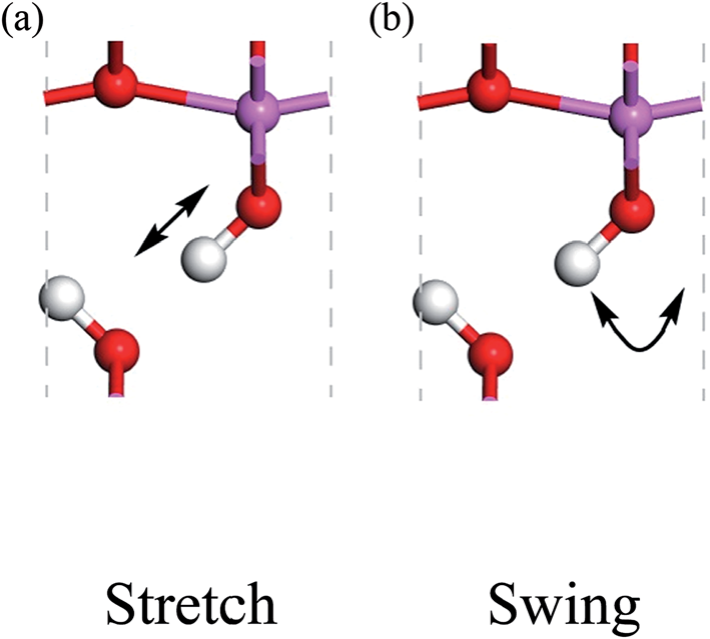
Proton-transfer modes in boehmite, with the direction of motions indicated by the arrows for the (a) stretch mode, (b) swing mode.

Because the three structures have almost the same thermodynamic stability, a boehmite sample may have domains of the three probable structures. The next question is whether they can transform into one another. If the structural transformations are easy at certain conditions, then the domains should be dynamic or volatile, otherwise the domains are static. We can assess energy barriers associated with the structural transformations, and compare the energy barriers with possible driving forces. Since the dipolar interactions between hydrogen-bond chains are weak, it is convenient for us to assume that only one hydrogen-bond chain changes and all other chains are constant. We study the transformation between the *Cmc*2_1_ and *Pmc*2_1_ structures, and propose two paths for hydrogen atoms to cause this structural transformation: the stretch mode and the swing mode (see Fig. 2). In the stretch mode, all the hydroxyl bonds break and hydrogen atoms move to nearby oxygen atoms to form new hydroxyl bonds (Fig. 2a). In the swing mode, a hydrogen atom remains around a same oxygen atom, and the hydroxyl bond does not break but only changes its direction (Fig. 2b). Each mode requires cooperative motion of all hydrogen atoms in a whole hydrogen-bond chain, otherwise a high-energy state may occur that either has two hydrogen atoms bonded to one oxygen atom, or has a very small distance between two hydrogen atoms boned to two neighboring oxygen atoms. We point out that both modes can independently transform the structure between *Cmc*2_1_ and *Pmc*2_1_, but long-range transfer of hydrogen atoms is only possible with both modes cooperating in concert.

**Fig. 3:**
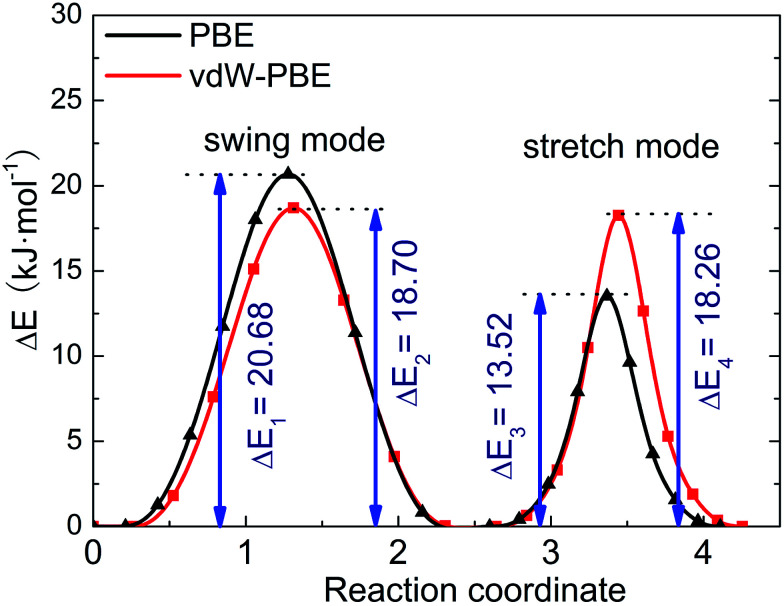
Energy barrier of proton-transfer processes in boehmite. The reaction of coordinates are scaled for comparison.

We use the CI-NEB method to calculate the energy barrier for the two hydrogen-transfer modes. As shown in Fig. 3, the PBE results indicate that the stretch mode has lower energy barrier (13.52 kJ mol^−1^) than the swing mode (20.68 kJ mol^−1^), while the vdW-PBE results show that the stretch mode and swing mode have almost the same energy barrier (18.70 and 18.26 kJ mol^−1^, respectively). The energy-barrier difference between these two modes is 7.16 kJ mol^−1^ for PBE and 0.44 kJ mol^−1^ for vdW-PBE, showing the influence the van der Waals forces on the transition states that involve hydrogen bonds in boehmite. Especially for the stretch mode, the energy barrier estimated by vdW-PBE is 5.18 kJ mol^−1^ higher than that by PBE. The large difference in energy barriers by the two methods can be understood by the arrangement of the atoms along the transition paths. In the stretch mode, the hydrogen atoms that are attached to one AlO_6_-octahedron layer move to another AlO_6_ layer, and this geometric change for the intermediate states is accompanied by energy increase by van der Waals interactions. In the swing mode, the hydrogen atoms are invariantly attached to one AlO_6_-octahedron layer along the whole transition path, for which the geometry change is smaller than that in the stretch mode.

Since the energy barriers are around 20 kJ mol^−1^, the energy required for transformation between the crystal structures can be provided by thermal fluctuations. Energy fluctuations at temperature *T* is roughly estimated to be *RT* (where *R* is gas constant, 8.31 J K^−1^ mol^−1^). Even though it is thermodynamically metastable relative to diaspore (α-AlOOH) under ambient conditions, boehmite can be stable at temperatures below its dehydration temperature, about 700–750 K, before transforming into γ-Al_2_O_3_.^45–47^ At *T* = 300 K, *RT* ≈ 2.5 kJ mol^−1^, and at the dehydration temperature (around 750 K), *RT* ≈ 6.2 kJ mol^−1^. In this temperature range (300–750 K), the energy barrier of the transition between *Cmc*2_1_ and *Pmc*2_1_ space groups is 3–8 times the *RT* value. It turns out that this structural transition is highly probable even at room temperature.

We can use the Arrhenius equation to calculate structural transition rate as^48^
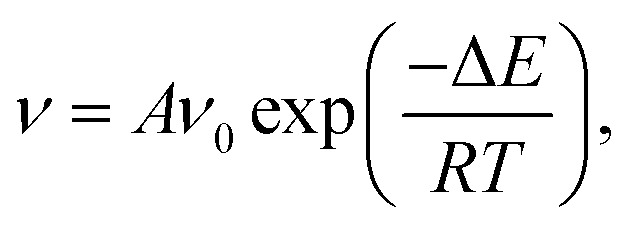
where *ν* stands for the frequency of successful structural transformation, *A* is a prefactor that is assumed to be a system-dependent constant, Δ*E* is the energy barrier for the transition, *R* is the gas constant, *T* is the temperature, and *ν*_0_ is the attempt rate, which corresponds to the frequency of a characteristic phonon mode. The proton-transfer modes involve coordinative motion of atoms, and certain phonon vibration modes may assist the processes if the motion of atoms in the phonon modes coincides with that in the proton-transfer modes. Tunega *et al.* have obtained boehmite crystal's phonon spectra,^48^ where two OH vibration modes match the stretch and swing modes of proton transfer in the present study. The value of *ν*_0_ for the stretch mode (2947 cm^−1^) is about three times that of the swing mode (1283 cm^−1^). Assuming that the prefactor *A* is the same for the two-transition modes, we can estimate the relative rate of the two transition modes. At room temperature (approximately 300 K), for the energy barriers calculated using PBE,
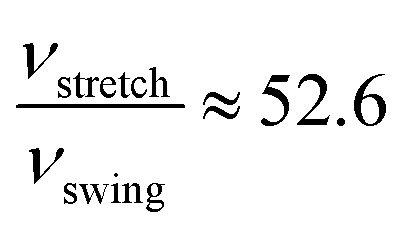
and for the vdW-PBE case:
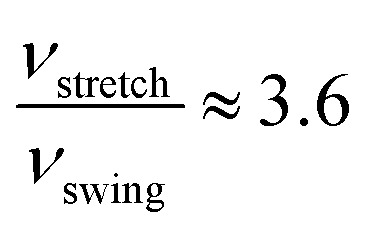


In both PBE and vdW-PBE calculations, the rate of stretch-mode transitions is higher than swing-mode transitions. But, the rate differences and the causes are quite different in PBE and vdW-PBE calculations. In PBE, the rate of stretch-mode transitions is several tens of times of swing-mode transitions, and the difference is mainly caused by its smaller energy barrier; while in vdW-PBE, the rate of stretch-mode transitions is only about four times that of swing-mode transitions, and the difference is mainly caused by the characteristic phonon frequency.

### 3.2 Crystal structure with defects of hydrogen vacancy

**Fig. 4:**
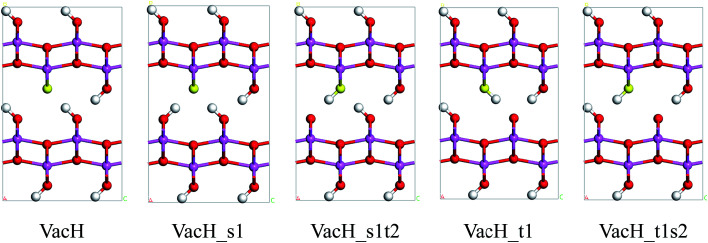
Five states along proton-transfer paths in boehmite containing a hydrogen vacancy: VacH, VacH_s1, VacH_s1t2, VacH_t1 and VacH_t1s2. The yellow atom marks the position of the oxygen atom to which the missing hydrogen atom is bonded.

Real boehmite crystals always contain various forms of defects, among which we concentrate on hydrogen vacancies. The normally continuous hydrogen-bond chains in a perfect lattice break at hydrogen vacancies, splitting into shorter chains. The hydrogen vacancies are expected to interrupt transition of nearby hydrogen atoms. In this study, we create hydrogen vacancies by removing a hydrogen atom from a supercell of boehmite, then design paths for hydrogen transfer to or from this vacancy position, and calculate energy barriers associated with these transition paths. Along the transition paths there are intermediate states for which the hydrogen atom in motion resides at one of the two equilibrium positions around an oxygen atom. In Fig. 4 we present five intermediate states, namely VacH, VacH_s1, VacH_s1t2, VacH_t1 and VacH_t1s2. Two paths can be composed by connecting subsets of these states for transferring a hydrogen atom along the [001] or [001̄] direction. The first path is from VacH to VacH_s1 *via* a swing-mode motion of a nearby hydrogen atom, and to VacH_s1t2 *via* another stretch-mode motion of the hydrogen atom. By repeating this path, the hydrogen-vacancy moves continuously towards the [001̄] direction and, equivalently, the hydrogen atoms in this hydrogen-bond chain move toward the [001] direction. The second path is from VacH to VacH_t1 *via* a stretch-mode motion of a nearby hydrogen atom, and to VacH_t1s2 *via* another swing-mode motion. The motion of the hydrogen vacancy and hydrogen atoms are both opposite to those in the first path.

**Fig. 5:**
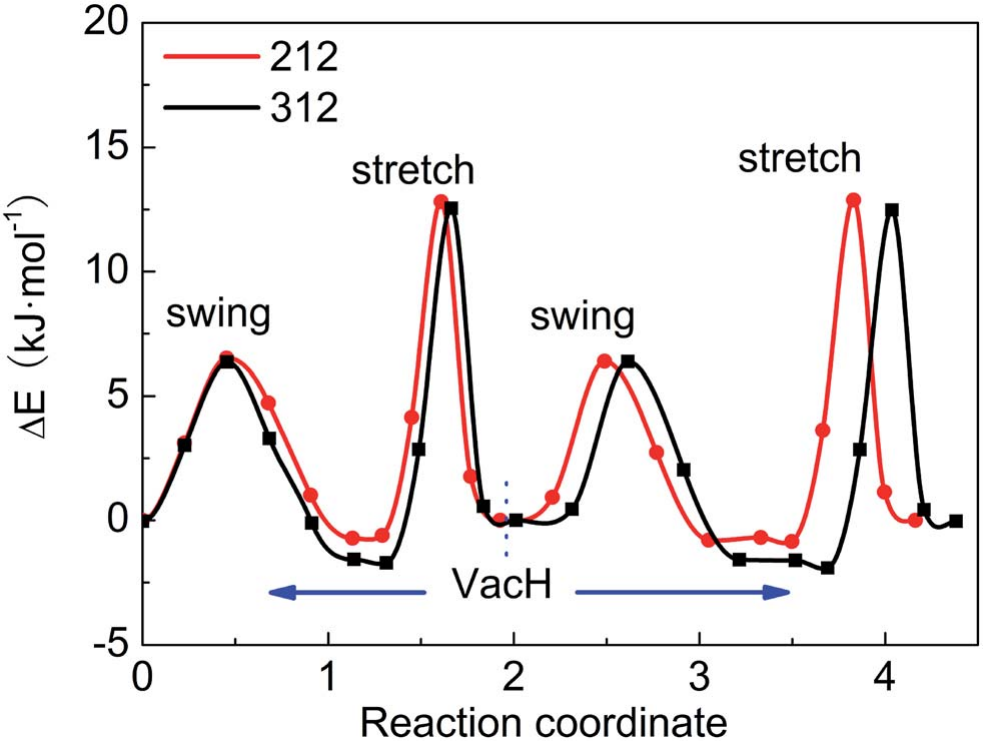
Energy barriers for hydrogen-transfer path from state VacH_t1s2 to VacH_t1, VacH, VacH_s1, then to VacH_s1t2, in supercell 2 × 1 × 2 and 3 × 1 × 2 using the vdW-PBE method.

These four steps involved in the two paths have different energy-barriers. We have used the CI-NEB method to calculate these energy-barriers and present the results in Table 3 and Fig. 5. We can clearly see that the two paths have very similar highest energy barriers in both PBE and vdW-PBE calculations. From our PBE calculations, the swing-mode steps, *i.e.* from VacH to VacH_s1 and from VacH_t1 to VacH_t1s2, have higher energy barriers than the stretch-mode steps, *i.e.* from VacH to VacH_t1 and from VacH_s1 to VacH_s1t2. The differences in these energy barriers are 1–2 kJ mol^−1^. For vdW-BPE calculations, the stretch-mode steps have higher energy-barriers than the swing-mode steps by approximately 6–8 kJ mol^−1^. The reasoning on the effects of vdW interactions on the stretch- and swing-modes has been discussed in the previous section for the perfect crystal, and also applies for the structures with hydrogen vacancies. The effects of hydrogen vacancies on the energy-barriers will be discussed in the next paragraph.

**Table 3:** The energy barriers of proton transfer processes. Unit: kJ mol^−1^

Calculation method	Super cell size	Process			
		VacH_t1 to VacH_t1s2	VacH to VacH_t1	VacH to VacH_s1	Vach_s1 to VacH_s1t2
PBE	2 × 1 × 2	9.20	8.12	9.13	7.70
	3 × 1 × 2	9.71	8.08	9.31	8.19
vdW-PBE	2 × 1 × 2	7.24	12.81	6.40	13.58
	3 × 1 × 2	7.92	12.54	6.38	14.09

**Fig. 6:**
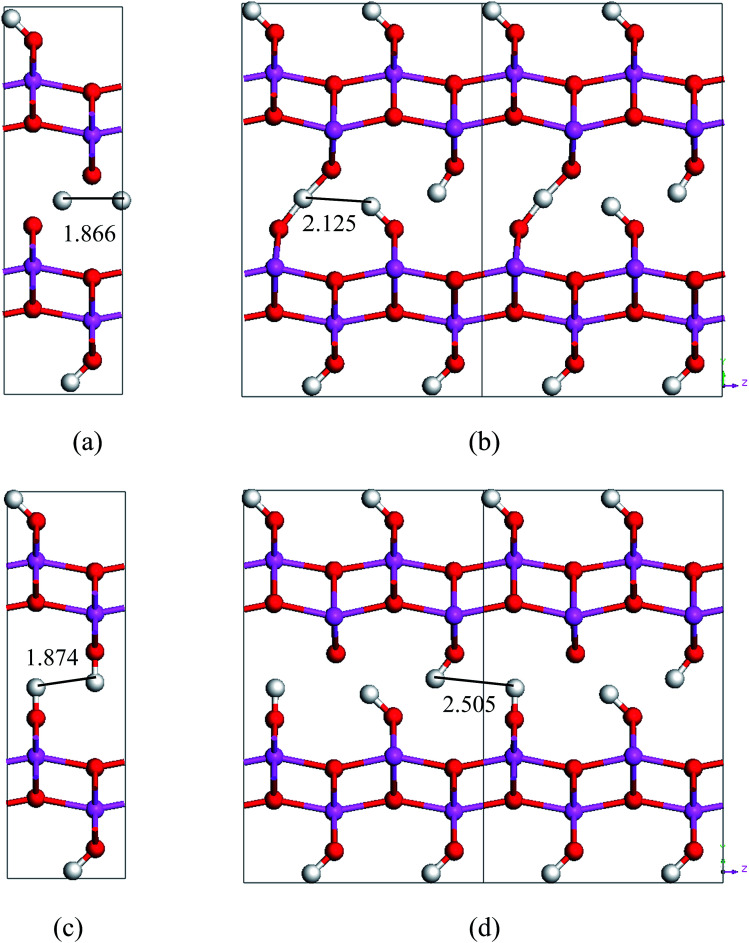
Saddle-point structures in the transition paths and lengths tween neighboring hydrogen atoms in these structures. (a) is for the stretch-mode transition from *Cmc*2_1_ to *Pmc*2_1_, (b) is for the stretch-mode transition from VacH_s1 to VacH_s1t2, (c) is for the swing-mode transition from *Cmc*2_1_ to *Pmc*2_1_, and (d) is for the swing-mode transition from VacH to VacH_s1. The sizes of the supercells in (c) and (d) are 2 × 1 × 2. The numbers are in unit of Å.

By comparing the energy-barriers in Fig. 3 and Table 3, one finds that the hydrogen vacancy lowers energy-barriers of both stretch- and swing-modes, but to different extents. The energy-barrier of the stretch mode is lowered by 4–6 kJ mol^−1^, and that of the swing mode is lowered by 10–12 kJ mol^−1^, both consistently in PBE and vdW-PBE calculations. The lowering in the energy barriers can be explained as follows. In a perfect crystal, all hydrogen atoms in a hydrogen-bond chain can only move coordinately to avoid the otherwise high-energy states. In the proximity of a hydrogen-vacancy, a hydrogen atom can move independently of other hydrogen atoms in either stretch or swing mode, requiring less energy than the motion of many hydrogen atoms in a perfect crystal. As for the difference in the vacancy-induced decreases of energy-barriers between the two modes (stretch and swing), it can tentatively be explained from the change in electrostatic interactions between the hydrogen ions. We assume the hydrogen ions carry the same amount of positive charge in a perfect crystal and vacancy-containing structures, thus the electrostatic interactions depend solely on distances between the ions. For simplicity, we only consider the electrostatic interaction between a hydrogen ion and its nearest neighbor. Along each transition path the state of the highest energy, or the saddle point, can be identified, and some representative ones are shown in Fig. 6. For the saddle-point structures in the two stretch-mode transition paths, the distance between the nearest neighboring hydrogen ions changes from 1.866 Å in a perfect crystal (Fig. 6a) to 2.125 Å in the vacancy-containing structure (Fig. 6b). For the two swing-mode transition paths, the distance between the nearest neighboring hydrogen ions changes from 1.874 Å in a perfect crystal (Fig. 6c) to 2.505 Å in the vacancy-containing structure (Fig. 6d). Starting from similar distances between nearest neighboring hydrogen ions, the larger increase in the H–H distances in the swing-mode steps leads to larger decrease in the associated energy barriers.

We have tested two sizes of supercells, namely 2 × 1 × 2 and 3 × 1 × 2, in calculating the energy barriers in the vacancy-containing structures that, similar to many other defect-containing structures, are expected to be sensitive to the supercells' sizes in the periodic boundary setting. For both PBE and vdW-PBE calculations, the differences between the energy-barriers in the two supercells are below 1 kJ mol^−1^, as can be derived from the numbers in Table 3. The trends of the energy barriers with respect to the steps are also consistent (see Fig. 6). We are confident that the calculated energy barriers are converged with respect to the supercell's size at 2 × 1 × 2.

## 4. Conclusions and remarks

In this paper, our computational results are mainly about thermodynamic stability of three probable structures of boehmite, and energy barriers for hydrogen transfer in boehmite's lattice with and without hydrogen vacancies. The results show that the *Pca*2_1_ structure has slightly lower total energy than the *Cmc*2_1_ and *Pmc*2_1_ structures due to different polarities of the OH bonds. Transfer paths of hydrogen atoms along the hydrogen-bond chains can be divided into steps of swing- and stretch-mode transitions, and are associated with different energy barriers that depend on computational settings. For a perfect crystal the energy barriers are below approximately 21 kJ mol^−1^, and for the structures with hydrogen vacancies, the energy barriers are below approximately 14 kJ mol^−1^. The computational settings of PBE and vdW-PBE lead to different orders of the energy barriers for swing-mode transitions and for stretch-mode transitions, in both perfect crystals and vacancy-containing structures. It has been observed van der Waals forces are important for hydrogen-bonds,^49^ thus the results of vdW-PBE should be more reliable and closer to reality.

The energy barriers are generally low in comparison with thermal fluctuation energies at room temperature and above, suggesting high mobility of hydrogen atoms in boehmite's lattice. Thus, the crystal structure of boehmite may comprise of all the three structures that are frequently changing into one another. It is difficult to identify domains of different polarity of the OH bonds without lowering the temperature. The average structure belongs to the *Cmcm* space group that is repeatedly confirmed by multiple experiments.

We suggest, based on the low energy barriers, that boehmite has a high proton conductivity that may be utilized for proton-conducting applications. The proton transfer mechanism in boehmite do not rely on liquid-phase water, and the working temperature can be elevated to above 100 °C. It has been stated in ref. 50 and 51 that a working temperature of 120 °C and 50% or lower relative humidity are targets for the development for automotive use of hydrogen/air fuel cells. The only drawback is that the proton conductivity of boehmite is highly anisotropic. Synthesizing and assembling highly ordered boehmite crystals are challenging, but it should be much easier to make boehmite filler materials for organic membranes to enhance their high-temperature proton conductivity.

## Conflicts of interest

There are no conflicts to declare.

## Supplementary Material
